# Material-Level Shrinkage and Tensile-Performance Screening of High-Strength Polyethylene-Fibre Engineered Cementitious Composite (PE-ECC) Mortar: MgO Versus Liquid Shrinkage-Reducing Admixture

**DOI:** 10.3390/polym18212798

**Published:** 2026-11-09

**Authors:** Qi Lu, Xian Zhou, Xian Wu, Zeyu Fan, Peihang Li, Wei Han

**Affiliations:** 1 Changjiang River Scientific Research Institute, Wuhan 430010, China; luqi3492@163.com (Q.L.); fanzeyu717728@163.com (Z.F.);; 2 Wuhan Municipal Engineering Design & Research Institute Co., Ltd., Wuhan 430023, China

**Keywords:** high-strength polyethylene-fibre engineered cementitious composites, material-level mixture selection, drying shrinkage, MgO expansive agent, shrinkage-reducing admixture, strain hardening

## Abstract

High-strength polyethylene-fibre engineered cementitious composites (PE-ECCs) provide tensile strain hardening and distributed cracking, but their high paste content and low water-to-binder ratio produce substantial shrinkage. This study conducted a multi-criteria screening of an MgO expansive agent and a shrinkage-reducing admixture (SRA) in a PE-ECC repair mortar for hydraulic-concrete applications. The reference matrix contained 65% P·II 52.5 cement, 10% silica fume, 25% blast-furnace slag, a water-to-binder ratio of 0.27, and 2.5 vol.% PE fibre. Drying shrinkage, compressive and flexural strengths, tensile response, crack number, and strain-based crack-opening estimate were assessed. The reference mortar reached 90.1 MPa compressive strength and 6.6% tensile strain but developed 1489 με of 28 d shrinkage. 2% MgO reduced the 28 d shrinkage to 1384 με, whereas higher dosages shifted from contraction to net expansion and reduced tensile performance. The liquid SRA reduced 28 d shrinkage to 982, 930, and 874 με at 1%, 2%, and 3%, respectively. The 1% mixture retained 82.3 MPa compressive strength, 5.7 MPa tensile strength, 4.4% tensile strain, and a crack-opening estimate of 66.4 μm. Under the adopted criteria, 1% SRA provided the most balanced combination of dimensional stability, strength, and tensile ductility, making it a candidate for application-specific evaluation in hydraulic-concrete repair.

## 1. Introduction

The global construction industry is undergoing a critical transition toward low-carbon, durable, and sustainable material systems, in which advanced polymer-modified cementitious composites have emerged as a core research direction for next-generation green building and infrastructure materials. The development of high-performance, dimensionally stable, and eco-friendly polymer-reinforced repair mortars is essential to extend the service life of hydraulic infrastructure, reduce recurrent maintenance costs, and lower the whole-life carbon footprint of construction projects. Concrete used in water-conveyance tunnels, culverts, channels, drainage structures, and other hydraulic facilities is consistently subjected to coupled deterioration factors, including moisture gradients, cyclic hydraulic loading, substrate deformation, and localized chemical attack [1,2]. In such harsh hydraulic service environments, conventional repair materials fail to meet the dual requirements of long-term structural durability and low-carbon sustainability due to their poor dimensional stability, brittle cracking, and short service life. Accordingly, high-performance repair overlay materials for hydraulic concrete require much more than high compressive strength alone; their volumetric stability, crack resistance, and service durability govern the long-term safety and sustainability of repaired infrastructure systems. Excessive shrinkage of thin repair overlays induces severe tensile stresses within the repair layer and at the interface with the parent concrete, whereas insufficient tensile deformation capacity causes stress localization, resulting in sparse, wide, penetrating cracks [3,4]. These defects not only degrade the mechanical performance of repaired structures but also exacerbate the ingress of water and corrosive media, accelerating material aging and increasing carbon emissions associated with frequent repairs. Therefore, dimensional compatibility and uniform crack control have become pivotal criteria for the sustainable selection and design of hydraulic concrete repair materials.

Engineered cementitious composites (ECCs), also known as strain-hardening cementitious composites, represent a class of advanced polymer-reinforced sustainable construction materials, uniquely designed to sustain progressive tensile loading after initial cracking and to distribute structural deformation through dense fibre-bridged microcracks [5,6]. Benefiting from the synergistic optimization of the cementitious matrix and polymer fibre reinforcement, high-strength ECC achieves a compressive strength exceeding 80 MPa and a tensile strain capacity of several percent through precise regulation of matrix fracture resistance and fibre-bridging efficiency [5,6,7]. As high-performance sustainable polymer reinforcement materials, polyethylene (PE) fibres possess superior tensile strength and elastic modulus, outstanding chemical stability, and excellent matrix compatibility, making them uniquely suitable for modifying high-strength cementitious matrices to construct durable, low-deformation composite systems [7,8]. Compared with traditional rigid repair materials, PE-ECC exhibits active crack dispersion rather than passive brittle failure, and the refined microcrack distribution effectively reduces the hydraulic connectivity of localized fractures in repair layers [9]. This excellent crack control and deformation compatibility endow PE-ECC with superior long-term durability in hydraulic environments, laying a solid foundation for developing low-maintenance, low-carbon sustainable repair materials for hydraulic infrastructure.

Nevertheless, the high binder dosage, fine aggregate gradation, and coarse-aggregate-free matrix design that endow high-strength PE-ECC with superior mechanical performance and toughness also render the material more susceptible to volumetric shrinkage deformation, which has become a key bottleneck restricting its large-scale sustainable engineering application. The drying shrinkage of high-performance ECC is generally higher than that of conventional concrete, and its deformation characteristics are governed by multiple factors, including water-to-binder ratio, supplementary cementitious materials, aggregate content, curing regime, and polymer fibre system [10,11,12]. In low water-to-binder high-strength matrices, internal moisture loss and the self-desiccation effect generate significant capillary tensile stresses, triggering continuous shrinkage and volumetric contraction. To address this issue, functional admixtures such as expansive agents and shrinkage-reducing admixtures (SRAs) have become key technical measures for shrinkage compensation and volumetric stability regulation in sustainable polymer cementitious composites. Effective shrinkage control technology can balance the reduction of matrix contraction, overall dimensional stability, and the micromechanical strain-hardening characteristics of polymer fibre-reinforced systems, which is critical to realizing the high durability and low-carbon service advantages of advanced PE-ECC repair materials in hydraulic engineering.

Expansive admixtures and liquid SRAs regulate volumetric deformation of polymer-modified cementitious composites through fundamentally different microscopic mechanisms, and their application exerts distinct effects on the mechanical properties, tensile toughness, and service durability of high-strength ECC systems. Relevant studies are listed in Table 1. These studies have explored the synergistic effects of expansive agents, SRAs, and mixture parameters on the performance of traditional PVA-ECC [10], and have evaluated the engineering applicability of SRAs in low-fibre sprayed hybrid ECC and ultra-high-performance cementitious matrices [13,14]. A limited number of comparative studies have analyzed the performance differences between an MgO expansive agent and an SRA in PVA-based strain-hardening cementitious composites, combining macroscopic mechanical tests with microscopic XRD and SEM characterization [15]. Relevant research on high-strength cementitious matrices also confirms that the dosage, hydration kinetics, and curing conditions of MgO govern the trade-off between shrinkage compensation and mechanical durability of composite materials [16,17,18]. However, systematic multi-criteria comparative research on the shrinkage control performance, mechanical stability, and flowability of high-strength PE-ECC regulated by an MgO expansive agent and a liquid SRA is still lacking.

**Table 1:** Scope distinction between representative shrinkage-control studies and the present work.

Reference	Fibre	w/b	Matrix/Focus	Control Route	Direct Tension	Crack Evaluation	Application Scope
Gao et al. [10]	PVA	0.25–0.33	Conventional ECC; strength varied	Low-alkali EA + SRA	Yes	Restrained slab cracking	No
Yuan et al. [15]	PVA, 2 vol.%	0.30	FA-rich SHCC with coarse silica sand	MgO vs. SRA	Yes	No crack-count/width dataset	Pavement focus
Arce and Sharifi [13]	Hybrid PVA/PE; low fibre	—	Practical spray-oriented ECC	SRA within multi-factor study	Yes	—	Yes; feasibility study
Present study	PE, 2.5 vol.%	0.27	90.1 MPa reference	MgO vs. liquid SRA	Yes	Crack count + strain-based opening	Intended hydraulic repair; material scale

This study provides a material-level stepwise screening dataset. Through a systematic examination of the multi-criteria shrinkage control effects of magnesium oxide (MgO) and liquid SRA on high-strength PE-ECC repair mortar, it clarifies the mechanism of their influence on volumetric deformation, tensile toughness, and fresh-state performance. The aim is to provide a feasible technical path for the design and engineering application of advanced, durable, and low-carbon polymer infrastructure materials in water hydraulic concrete repair. Due to the sensitivity of the selection process to the preset threshold and the influence of unverified low-dose mixtures, the recommended mixture is regarded as a candidate for further evaluation rather than an already verified actual construction formula.

## 2. Materials and Methods

### 2.1. Raw Materials

**Figure 1:**
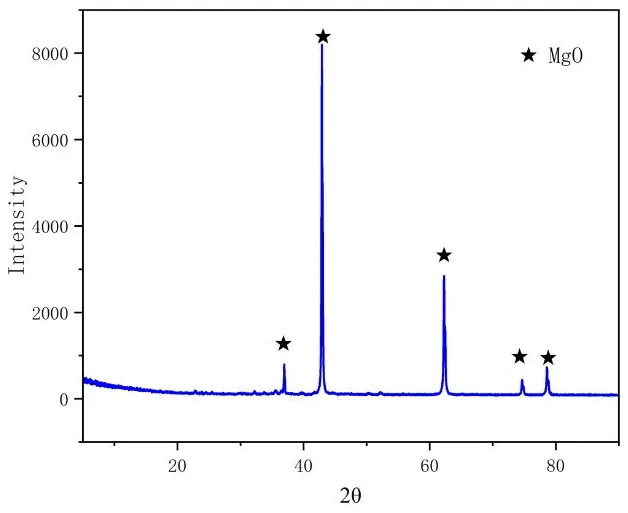
XRD of MgO.

**Figure 2:**
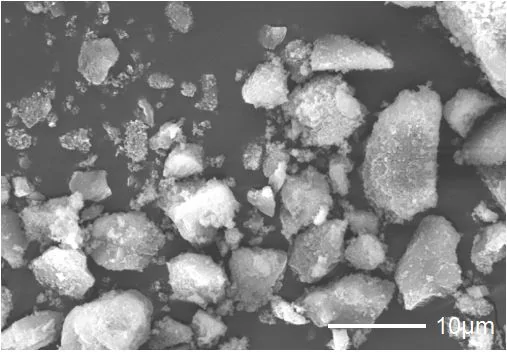
SEM of MgO.

MgO was produced by Jiangsu Sobute New Materials Co., Ltd., Nanjing, China. The chemical composition and selected performance indicators of MgO are shown in Table 2. The reactive time measured by the citric acid reaction method is 100 s. Its performance meets the requirements of CBMF 19-2017 [19]. The XRD of the MgO is shown in Figure 1. In addition to MgO, it also contains small amounts of minerals such as f-CaO, magnesite, quartz, talc, and other minerals. As can be seen in the SEM image of the MgO (Figure 2), the particles are composed of many fine MgO grains, which are granular and irregular in shape.

**Figure 3:**
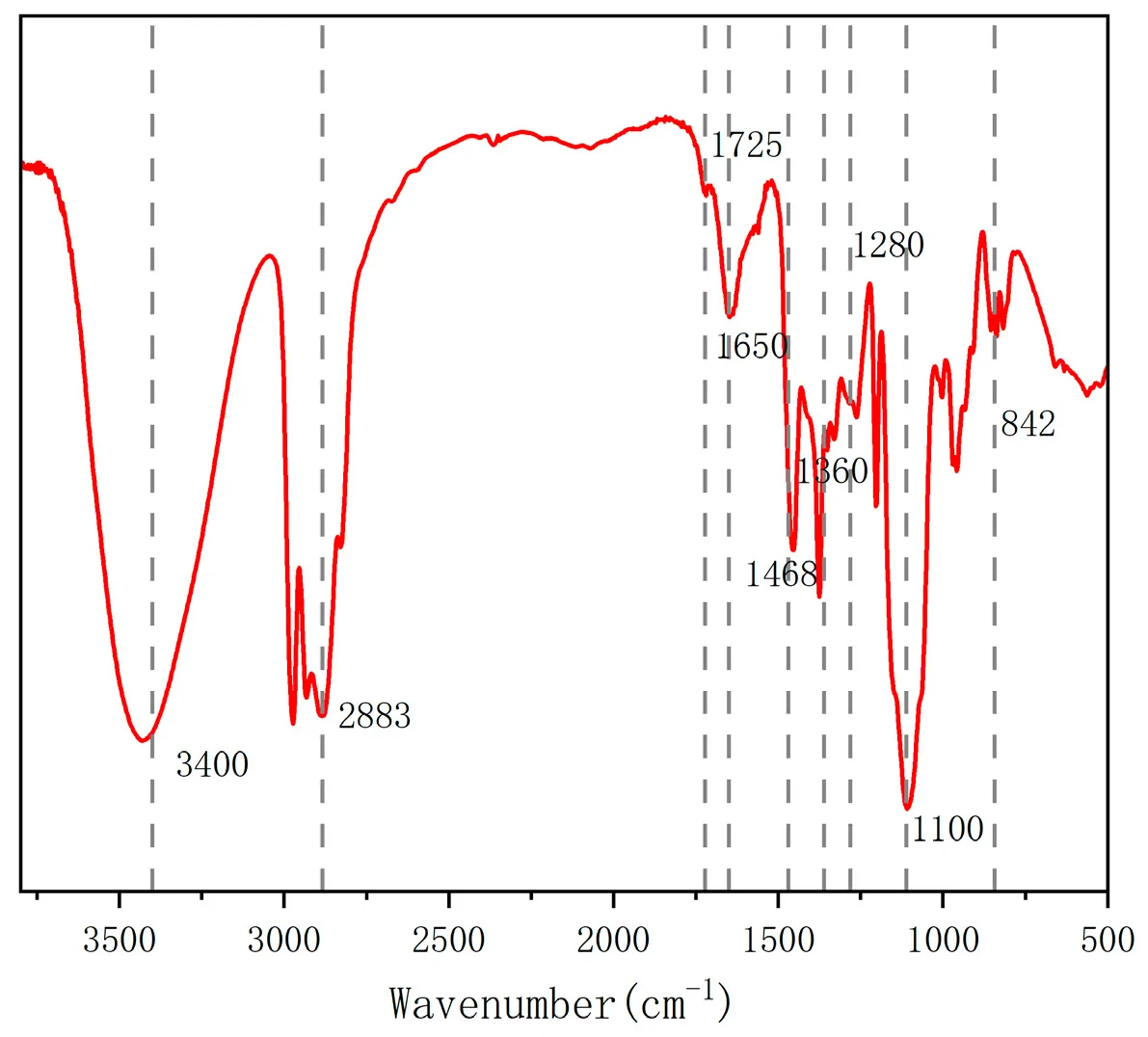
FTIR spectrum of SRA.

SRA was produced by Shanghai Devino Advanced Material Technology Co., Ltd., Shanghai, China. Its appearance is a colorless to light yellow liquid, with a pH value of 7–11 and a density of 1.013 g/cm^3^. As shown in Table 3, the relevant properties meet the requirements for standard-type shrinkage-reducing agents in JC/T 2361-2016 [20]. The ATR-FTIR spectrum of the SRA is shown in Figure 3. Characteristic absorption bands were observed at approximately 3400 cm^−1^ (O–H stretching), 2870~2930 cm^−1^ (C–H stretching), 1725 cm^−1^ (C=O stretching), and 1100 cm^−1^ (C–O–C stretching), indicating the presence of hydroxyl (–OH) groups (including free water in the commercial product), ether (C–O–C) linkages, and a minor carbonyl functionality in the active components. These features suggest that the SRA is likely a polyether/polyol-type compound, although the full commercial formulation was not disclosed by the supplier.

As shown in Table 4, the binder consisted of P·II 52.5 Portland cement, silica fume, and ground granulated blast-furnace slag. The cement had a density of 3.13 g/cm^3^ and a specific surface area of 377.2 m^2^/kg. The silica fume had a density of 2230 kg/m^3^, water-demand ratio of 113%, specific surface area of 17,600 m^2^/kg, and 28 d activity index of 116%. The S75 slag had a density of 2.90 g/cm^3^, specific surface area of 364.9 m^2^/kg, and 28 d activity index of 99%. Fine ground sand had a maximum particle size of 160 μm, an apparent density of 2.74 g/cm^3^, and a saturated-surface-dry absorption of 0.1%. The mixing water is tap water. A PCA polycarboxylate high-range water-reducing admixture was used at 1% of binder mass; its water-reduction ratio was 27.6%, air content 2.0%, chloride ion content 0.02%, and alkali content 0.63%. High-strength PE fibres were 12 mm long and approximately 25 μm in diameter, with a tensile strength of 3100 MPa, elastic modulus of 122 GPa, elongation at break of 3.5%, and density of 0.97 g/cm^3^.

**Table 2:** The chemical composition and selected performance indicators of MgO.

Name	Active Reaction Time (s)	Density (g/cm^3^)	Chemical Composition (%)				
			CaO	MgO	Fe_2_O_3_	Al_2_O_3_	SiO_2_
MgO	100	3.21	1.46	94.28	0.43	–	0.62

**Table 3:** Quality inspection results of the SRA.

Content		JC/T 2361-2016	Result
Density (g/cm^3^)		1 ± 0.02	1.013
Chloride ion content (%)		≤0.02	0.005
Air content (%)		≤5.0	3.3
Setting time (min)	Initial	≤+120	+23
	Final		+38
Compressive strength ratio (%)	7 d	≥90	109
	28 d	≥95	107
Shrinkage reduction rate (%)	7 d	≥35	45.4
	28 d	≥30	39.6
	60 d	≥25	35.2

**Table 4:** Physical properties and proportions of the principal constituents.

Constituent	Density	Fineness/Size	Other Property	Role or Dosage
P·II 52.5 cement	3.13 g/cm^3^	377.2 m^2^/kg	28 d cement strength 59.8 MPa	Primary binder
Silica fume	2.23 g/cm^3^	17,600 m^2^/kg	Water-demand ratio 113%; activity index 116%	10% of binder
S75 slag	2.90 g/cm^3^	364.9 m^2^/kg	28 d activity index 99%	25% of binder
Ground sand	2.74 g/cm^3^	Maximum size 160 μm	SSD absorption 0.1%	s/b = 0.40
PCA superplasticizer	Liquid	Water reduction 27.6%	Air 2.0%; Cl^−^ 0.02%; alkali 0.63%	1% of binder

### 2.2. Mixtures and Sequential Screening Design

The reference mixture H-3 contained 65% cement, 10% silica fume, and 25% slag by binder mass, with a sand-to-binder ratio of 0.40, w/b of 0.27, 1% superplasticizer, and 2.5 vol.% PE fibre. MgO dosages of 2%, 5%, 8%, 10%, and 20% were investigated. The mixture labels PZ-1, PZ-2, PZ-3, PZ-4, and PZ-5 follow the experimental sequence rather than ascending dosage. Liquid SRA dosages were 0.5%, 1%, 2%, and 3% of binder mass. The experimental program comprised a broad Stage I screening followed by a targeted Stage II tensile assessment.

#### 2.2.1. Stage I: Fresh-State, Dimensional, and Strength Screening

As shown in Table 5, Stage I established the dosage-response ranges of the two shrinkage-control routes. Flow diameter and drying shrinkage were used to identify mixtures with acceptable fresh consistency and stable dimensional behavior. Compressive and flexural strengths at 1, 7, and 28 d were then compared for the reference, the dimensionally stable MgO mixtures, and all SRA mixtures. The 10% and 20% MgO mixtures were not carried into strength or tensile evaluation after visible longitudinal bending developed during the length-change test.

#### 2.2.2. Stage II: Tensile and Crack-Control Assessment

Stage II examined whether shrinkage control preserved tensile strain hardening and distributed cracking. The SRA mixtures that met the 28 d contraction criterion (JS-2 to JS-4) were compared with H-3. PZ-2 and PZ-5 were also retained to quantify the tensile consequence of entering the MgO-induced net-expansion regime. PZ-1 and JS-1 were not advanced because their 28 d contractions remained above 1000 με. Since the 28-day shrinkage of PZ-1 and JS-1 still exceeded 1000 με, they were not included in the subsequent screening. Nevertheless, their tensile properties and cracking behavior were still tested to support the mechanistic analysis. Details are provided in Table 6.

**Table 6:** Stage II selection, measurements, and experimental purpose.

Decision Group	Mixtures	Stage I Basis	Stage II Measurements	Purpose
Reference	H-3	Benchmark response	Direct tension, *n* = 3; crack count, *n* = 6	Define tensile and cracking baseline
MgO transition	PZ-2, PZ-5	Net expansion at 28 d	Direct tension, *n* = 3; crack count, *n* = 6	Quantify tensile effects in expansion regime
SRA candidates	JS-2, JS-3, JS-4	28 d contraction ≤1000 με	Direct tension, *n* = 3; crack count, *n* = 6	Compare strain-hardening retention across SRA dosage
Not advanced	PZ-1, JS-1	1384 and 1265 με contraction	Direct tension, *n* = 3; crack count, *n* = 6	Above Stage I contraction criterion (not selected, supplementary tested)

### 2.3. Mixing, Casting, and Curing

Cement, silica fume, slag, ground sand, and the powdered modifier, where applicable, were dry-mixed to visual uniformity. Pre-weighed water, superplasticizer, and liquid SRA, where applicable, were added and mixed until a homogeneous matrix formed. PE fibres were introduced gradually at low speed, followed by high-speed mixing. Fresh mortar was placed in the relevant molds and compacted on a standard vibrating table for 120 s. Specimens were stored at 20 ± 2 °C and RH > 90% for 24 h, demoulded, and returned to standard curing until the specified test age.

### 2.4. Methods

#### 2.4.1. Flowability

Flowability was determined in accordance with GB/T 2419-2005 [21]. The freshly mixed mortar was placed into a standard truncated conical mold in two layers, compacted as specified in the standard, and then subjected to 25 jolts on the flow table within 25 ± 1 s. The flow diameter was taken as the average of two diametrically opposed readings, which constituted one measurement set, and three replicate sets were tested for each mixture. The entire test was completed within 6 min after the first water–cement contact.

#### 2.4.2. Drying Shrinkage and Length Change

Length change was measured following JC/T 603-2004 [22] using 25 mm × 25 mm × 280 mm prisms with an effective gauge length of 250 mm. Specimens were standard-cured for 1 d, demoulded, and measured to 0.001 mm to establish *L_0_*. They were then exposed at 20 ± 3 °C and 50 ± 4% RH. Measurements were scheduled at ages of 3, 7, 14, and 28 d from casting, corresponding to approximately 2, 6, 13, and 27 d of drying exposure after the initial measurement. Three prisms were tested for each mixture. The shrinkage strain was calculated as *(L_0_ − L_t_)*/250 and expressed in microstrain. For endpoint comparisons, a negative sign denotes contraction and a positive sign denotes expansion; shrinkage magnitudes are reported as positive absolute values.

#### 2.4.3. Compressive and Flexural Strength Test

Compressive and flexural strengths were determined in accordance with JC/T 2381-2016 [23]. Flexural specimens were 40 mm × 40 mm × 160 mm prisms, and compressive strength was determined on the prescribed 40 mm cubes. Tests were conducted at 1, 7, and 28 d, and the results are reported as group means.

#### 2.4.4. Direct Tension and Strain-Based Crack-Opening Estimate

Direct tension followed JC/T 2461-2018 [24]. Dumbbell-shaped plates had an 80 mm long and 30 mm wide constant-section region. Loading was displacement-controlled at 0.5 mm/min and terminated when load fell to 80% of the maximum. Three specimens were used per mixture, and the extensometer gauge length *L_tg_* was 80 mm.

Representative stress-strain curves were used to compare the tensile-response shape and sequential multiple-cracking behavior of the Stage II mixtures.

Cracks were counted along the centreline of both broad faces within the gauge region, and the mean crack number *N_c_* was calculated. Six specimens were used for the crack-count evaluation. The strain-based estimated mean crack opening, *w_est_* was calculated as:

*w_est_* = (*ε_tu_* × *L_tg_*/*N_c_*) × 1000

where ε_tu_ is the ultimate tensile strain expressed as a dimensionless decimal (for example, 0.066 rather than 6.6), L_tg_ = 80 mm, N_c_ is the mean crack number, and w_est_ is expressed in μm. If ε_tu_ is entered as a percentage, the numerator is divided by 100.

### 2.5. Data Processing and Performance Indices

Each mixture was tested three times for flowability. Compressive Strength, Flexural Strength, tensile strength and drying shrinkage used three specimens per mixture, and crack counting used six specimens per mixture. The three specimens for direct tension tests and the six specimens for crack counting were companion specimens from the same casting batch, rather than identical specimens. Crack counting was conducted on six independent specimens, whereas the tensile properties were derived from the average of three additional specimens. The calculated crack-opening values are group-level approximations, not direct measurements of individual crack widths. Mean values were used for between-mixture comparisons. Property retention was calculated as:

*R_i_* = *X_i_/X_0_* × 100%

where *X_i_* is a modified-mixture value and *X_0_* is the H-3 value.

For mixtures that remained in contraction, shrinkage reduction was calculated as:

*(ε_0_ − ε_i_)/ε_0_* × 100%

### 2.6. Performance Criteria for Multi-Criteria Selection

In Table 7, Six criteria were applied jointly to evaluate dimensional stability, mechanical resistance, tensile deformation capacity, and distributed cracking.

## 3. Results

### 3.1. Flow Response

**Figure 4:**
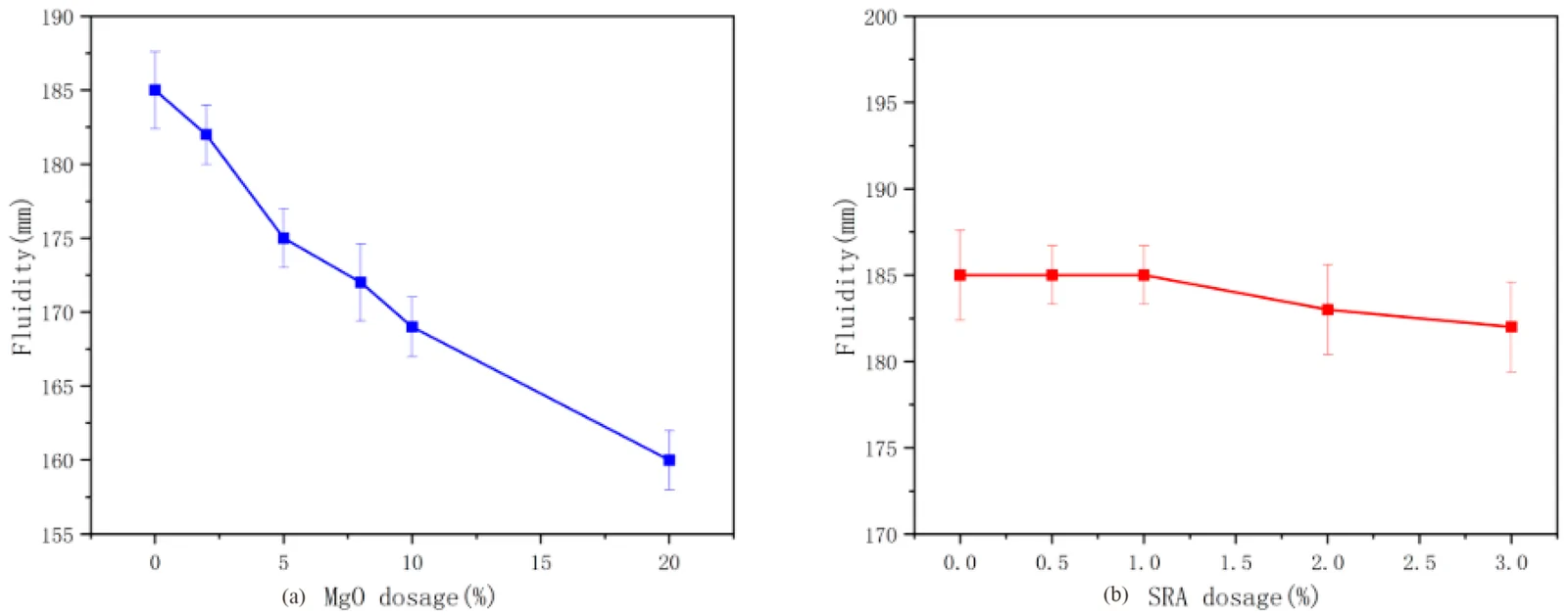
Effect of admixture dosage on the flowability of PE-ECC: (**a**) MgO and (**b**) SRA.

According to the flowability test results, the H-3 mixture without MgO or SRA exhibited a flow diameter of 185 mm. With increasing MgO dosage, the flow diameter showed a decreasing trend: at MgO contents of 2%, 5%, 8%, 10%, and 20%, the flow diameters were 182, 175, 172, 169, and 160 mm, respectively. In contrast, the SRA had only a minor effect on flowability. Based on the mean flow results, the flow diameter remained unchanged at 185 mm at SRA dosages of 0.5% and 1%; as the SRA dosage was further increased to 2% and 3%, the flow diameter decreased to 183 mm and 182 mm, respectively. These observations indicate that the incorporation of MgO reduced the flowability, whereas the SRA had a negligible influence on the flowability of the mixtures. Figure 4 highlights the difference in the dosage-response behavior between the two admixtures.

The detrimental effect of MgO on flowability may reflect changes in powder packing, water demand, early-age reactions, or compatibility with the polycarboxylate-based superplasticizer. According to the XRD and SEM results, the MgO used in this study consists of fine crystals with a loose and porous structure, and its specific surface area is considerably larger than that of cement particles. Moreover, the higher the MgO reactivity, the larger the specific surface area. As a result, the free water in the paste tends to be preferentially adsorbed on the surfaces of MgO particles, forming a water film around them. In addition, the Mg(OH)_2_ crystals formed at early ages are extremely fine and exhibit a certain water-absorbing capacity. These effects make it difficult for the flowability of the paste to be substantially improved. Furthermore, since the MgO was added as an external admixture, the effective water-to-binder ratio was reduced, which also contributed to the decrease in flowability [25].

In contrast, the SRA is essentially a surfactant. At low dosages, it may modify the viscosity, air-entrainment behavior, or wetting characteristics of the concrete [26]. However, in the present PE-ECC system, the water-to-binder ratio is relatively low (0.27), resulting in a relatively high yield stress of the paste; consequently, no change in flowability was observed at low SRA dosages. In addition, the SRA used in this study is polyether-type, whose surface activity is primarily aimed at lowering the surface tension of the pore solution rather than altering the flocculation state of the cementitious particles. Therefore, even when the SRA dosage was increased to 2–3%, its effect on the flowability of the paste remained limited. This is consistent with the findings of Zhang et al. [27], who reported that shrinkage-reducing admixtures have no significant influence on the slump or slump flow of concrete.

However, this does not mean that the SRA had no effect on the rheological properties. As a surfactant, the SRA may modify the viscosity, air-entrainment behavior, or wetting characteristics of the PE-ECC, but these changes may not necessarily result in noticeable differences in the flowability test indicators. H-3 had a flow diameter of 185 mm. Increasing MgO dosage reduced the diameter progressively to 182, 175, 172, 169, and 160 mm at 2%, 5%, 8%, 10%, and 20%, respectively. SRA had only a minor effect on the flowability of the mixtures. Based on the mean flow diameters, the flow diameter remained unchanged at 185 mm at SRA dosages of 0.5% and 1%. As the SRA dosage was further increased to 2% and 3%, the flow diameter decreased from 185 mm to 183 mm and 182 mm, respectively. Figure 3 highlights the contrast between the two dosage responses. The MgO trend may reflect changes in powder packing, water demand, early reaction, or compatibility with the polycarboxylate admixture. This does not imply that SRA has no effect on rheology. As a surfactant, SRA may alter the viscosity, air-entraining behavior, or wetting characteristics of PE-ECC, yet they do not necessarily lead to obvious changes in the fluidity test index. This is consistent with the findings of [28] et al.

### 3.2. Drying Shrinkage and Net Expansion

**Figure 5:**
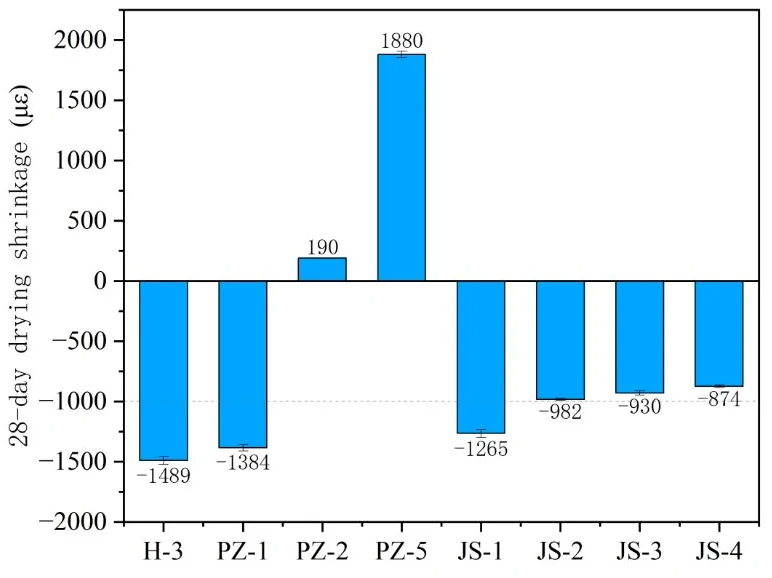
Net length-change endpoints at 28 d. Negative values denote contraction and positive values denote net expansion.

**Figure 6:**
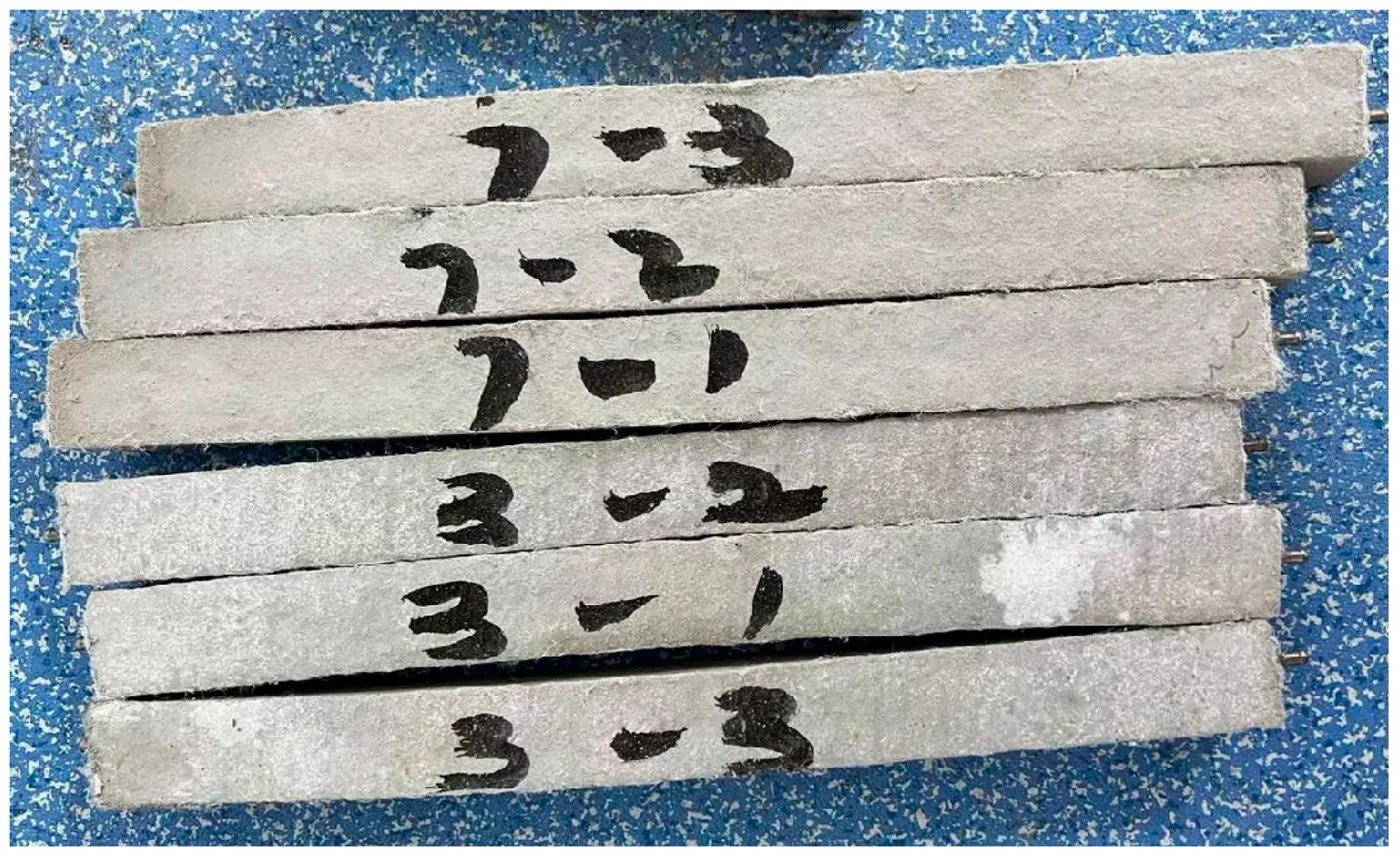
Photograph of the 10% and 20% MgO screening prisms at 28 d, showing visible longitudinal curvature.

As shown in Table 8, the 28 d shrinkage of PZ-1 was 1384 με, whereas PZ-2 and PZ-5 showed net expansions of 190 and 1880 με, respectively (Figure 5). PZ-5 (8% MgO) showed fine surface cracks and slight edge peeling after 28 days of drying, accompanying the large net expansion. PZ-3 (10% MgO) and PZ-4 (20% MgO) were excluded from the one-dimensional endpoint comparison after bending was observed, because curvature violates the assumption of uniform axial length change (Figure 6). The SRA series exhibited a monotonically decreasing trend in 28-day shrinkage. JS-1 (0.5% SRA) showed a shrinkage of 1265 με, which is 224 με lower than that of H-3. As the SRA dosage was further increased, the incremental benefit gradually diminished: increasing the dosage from 1% to 3% resulted in a further reduction of 108 με in shrinkage (from 982 to 874 με), but was accompanied by a significantly greater loss of tensile properties. A detailed discussion of the expansion and shrinkage characteristics is presented in the Section 4.

**Table 8:** Drying shrinkage and net expansion at 28 d.

Mixture	28 d Response (με)	Response Type	Comparison
H-3	1489 ± 32.5	Contraction	Reference response
PZ-1 (2% MgO)	1384 ± 25.3	Contraction	7.1% lower than H-3
PZ-2 (5% MgO)	190 ± 2.4	Net expansion	Transition from contraction to expansion
PZ-5 (8% MgO)	1880 ± 26.8	Net expansion	Pronounced expansion
PZ-3/PZ-4	—	Visible bending at 28 d	Dimensional instability at 10% and 20% MgO
JS-1 (0.5% SRA)	1265 ± 32.1	Contraction	15.0% lower than H-3
JS-2 (1% SRA)	982 ± 8.9	Contraction	34.0% lower than H-3
JS-3 (2% SRA)	930 ± 17.2	Contraction	37.5% lower than H-3
JS-4 (3% SRA)	874 ± 10.9	Contraction	41.3% lower than H-3

### 3.3. Compressive and Flexural Strength

**Figure 7:**
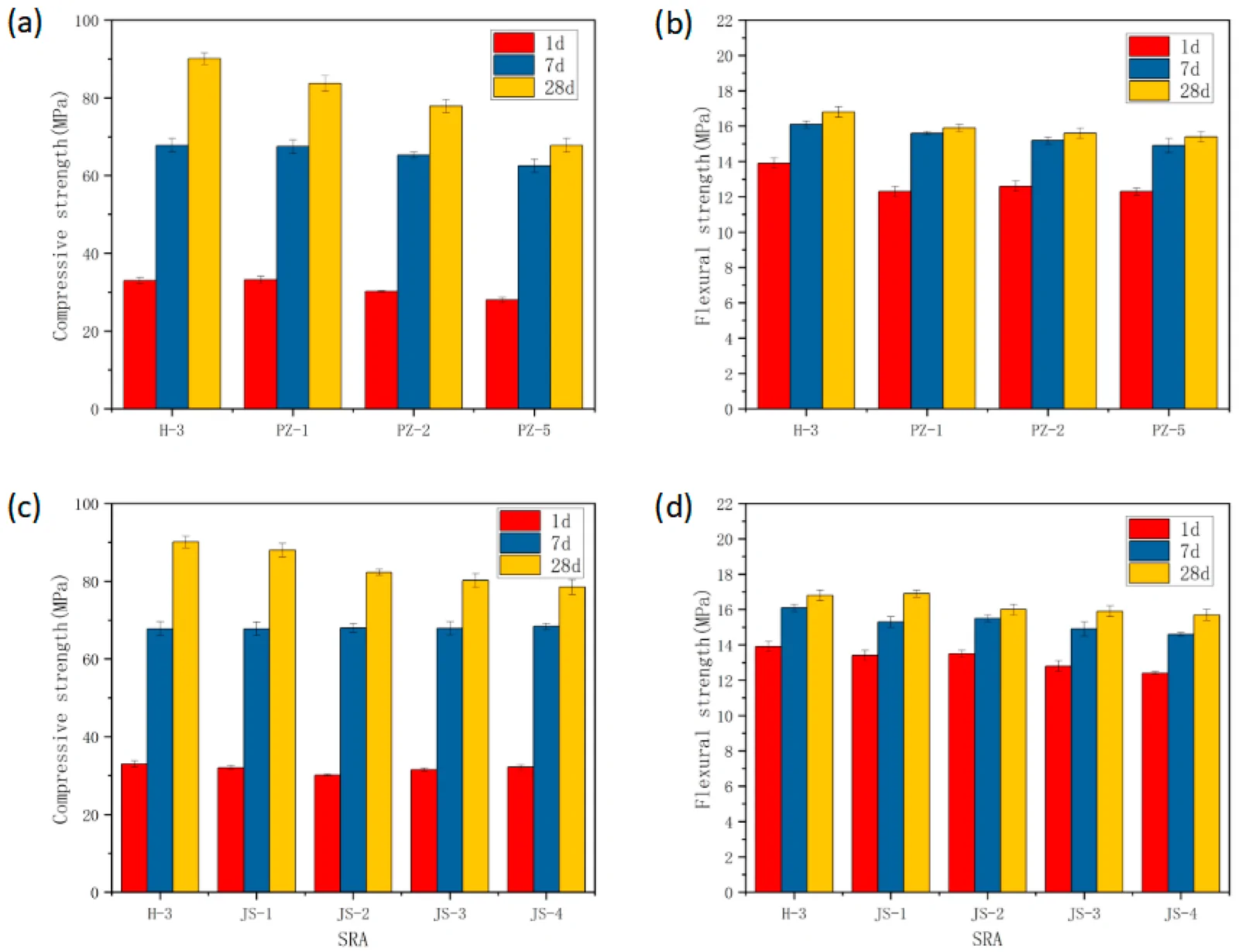
Strength development: compressive strength of (**a**) the MgO series and (**b**) the liquid SRA series, and flexural strength of (**c**) the MgO series and (**d**) the liquid SRA series. Dashed lines indicate the 28 d performance criteria. Values are group means.

In Table 9, H-3 reached 90.1 MPa in compression and 16.8 MPa in flexure at 28 d. The 28 d compressive strength decreased with increasing modifier dosage in both series. PZ-1, PZ-2, and PZ-5 retained 92.9%, 86.5%, and 75.2% of H-3, respectively; PZ-5 fell below the 70 MPa compressive-strength criterion. JS-1 to JS-4 retained 97.7%, 91.3%, 89.0%, and 87.1%, respectively. Flexural strength was less sensitive, and all evaluated mixtures remained above 15.4 MPa at 28 d. Figure 7 shows that most between-mixture separation emerged between 7 and 28 d in compression, whereas the flexural trajectories remained comparatively compact.

**Table 9:** Compressive and flexural strength development (MPa; group means).

Mixture	*f_c_*, 1 d (MPa)	*f_c_*, 7 d (MPa)	*f_c_*, 28 d (MPa)	*f_f_*, 1 d (MPa)	*f_f_*, 7 d (MPa)	*f_f_*, 28 d (MPa)
H-3	33.0 ± 0.8	67.8 ± 1.8	90.1 ± 1.5	13.9 ± 0.3	16.1 ± 0.2	16.8 ± 0.3
PZ-1	33.2 ± 0.9	67.5 ± 1.7	83.7 ± 2.0	12.3 ± 0.3	15.6 ± 0.1	15.9 ± 0.2
PZ-2	30.2 ± 0.3	65.3 ± 0.7	77.9 ± 1.7	12.6 ± 0.3	15.2 ± 0.2	15.6 ± 0.3
PZ-5	28.0 ± 0.7	62.5 ± 1.7	67.8 ± 1.8	12.3 ± 0.2	14.9 ± 0.4	15.4 ± 0.3
JS-1	32.0 ± 0.6	67.8 ± 1.7	88.0 ± 1.8	13.4 ± 0.3	15.3 ± 0.3	16.9 ± 0.2
JS-2	30.1 ± 0.3	68.0 ± 1.1	82.3 ± 0.8	13.5 ± 0.2	15.5 ± 0.2	16.0 ± 0.3
JS-3	31.5 ± 0.5	67.9 ± 1.7	80.2 ± 1.8	12.8 ± 0.3	14.9 ± 0.4	15.9 ± 0.3
JS-4	32.2 ± 0.6	68.4 ± 0.8	78.5 ± 1.9	12.4 ± 0.1	14.6 ± 0.1	15.7 ± 0.3

### 3.4. Direct Tensile Response and Distributed Cracking

**Figure 8:**
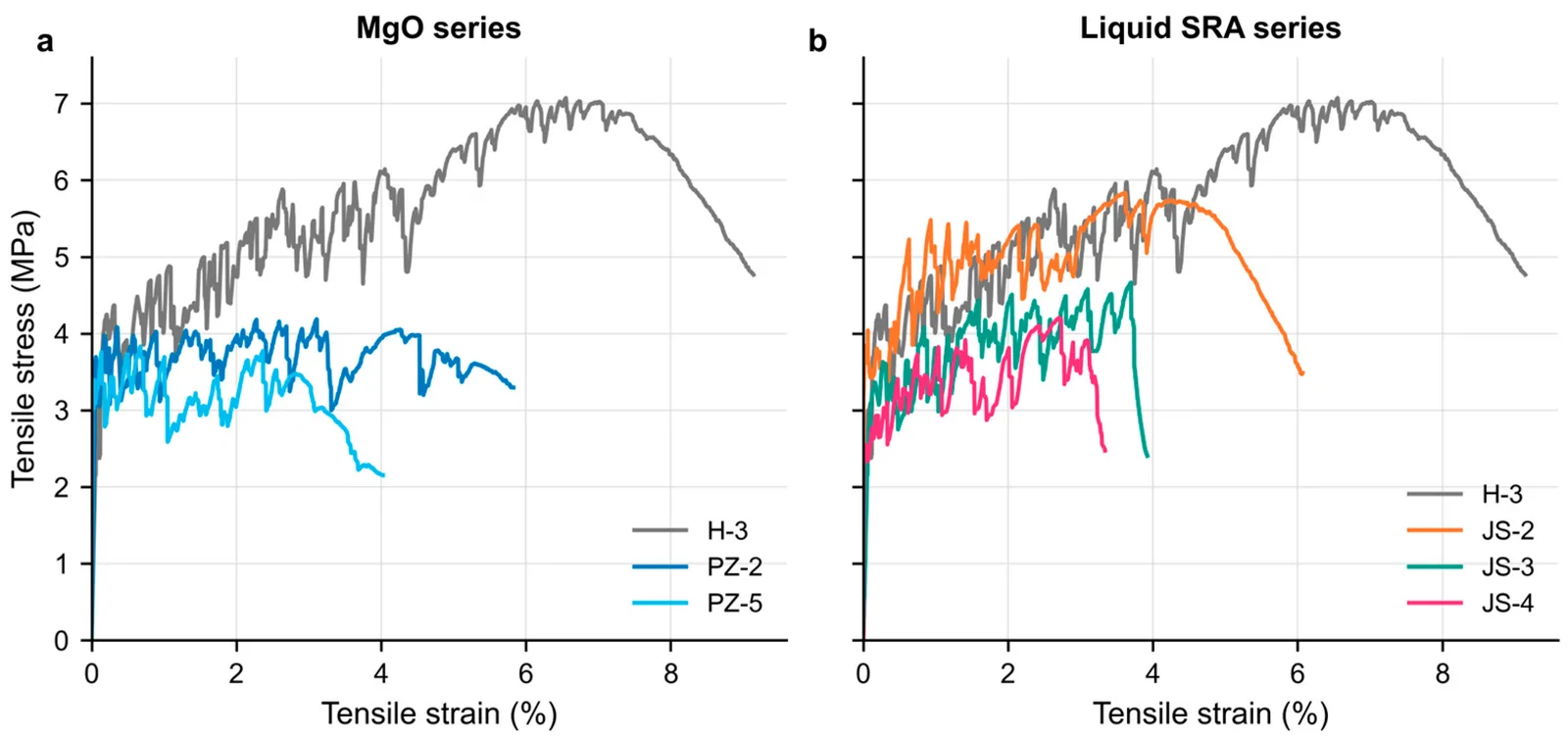
Representative uniaxial tensile stress-strain curves for (**a**) the MgO series and (**b**) the liquid SRA series.

**Figure 9:**
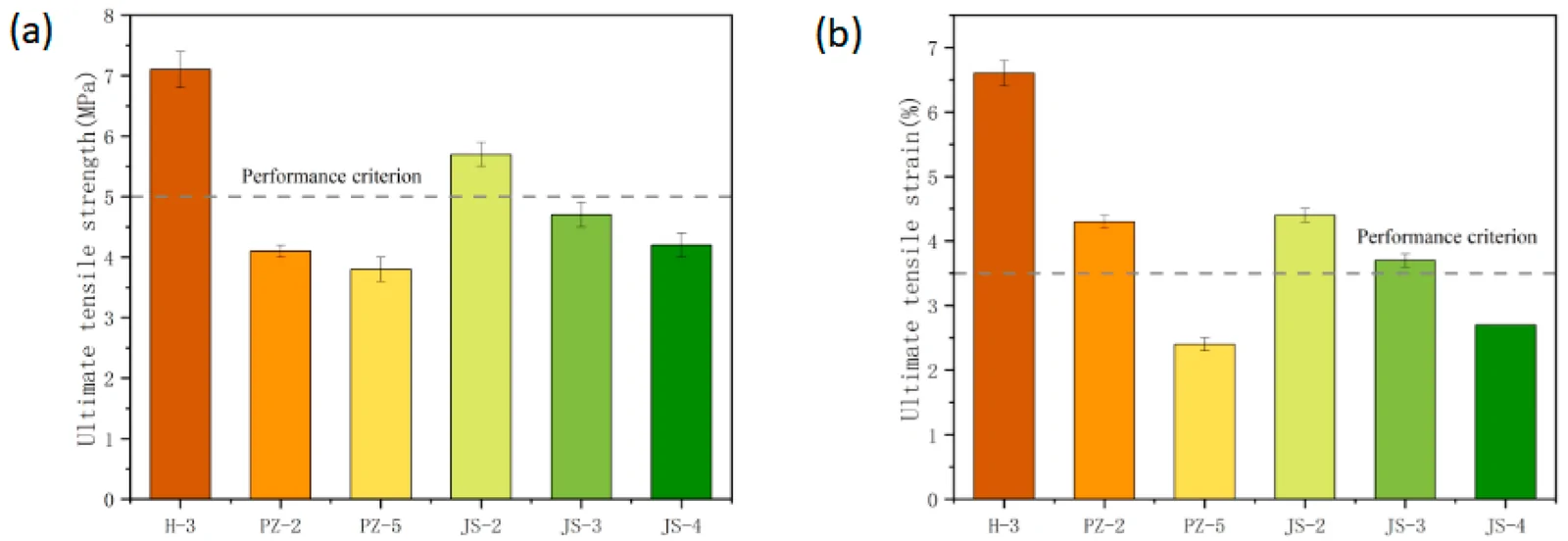
Direct tensile response of the Stage II mixtures: (**a**) ultimate tensile strength and (**b**) ultimate tensile strain. Dashed lines show the performance criteria. Values are group means.

**Figure 10:**
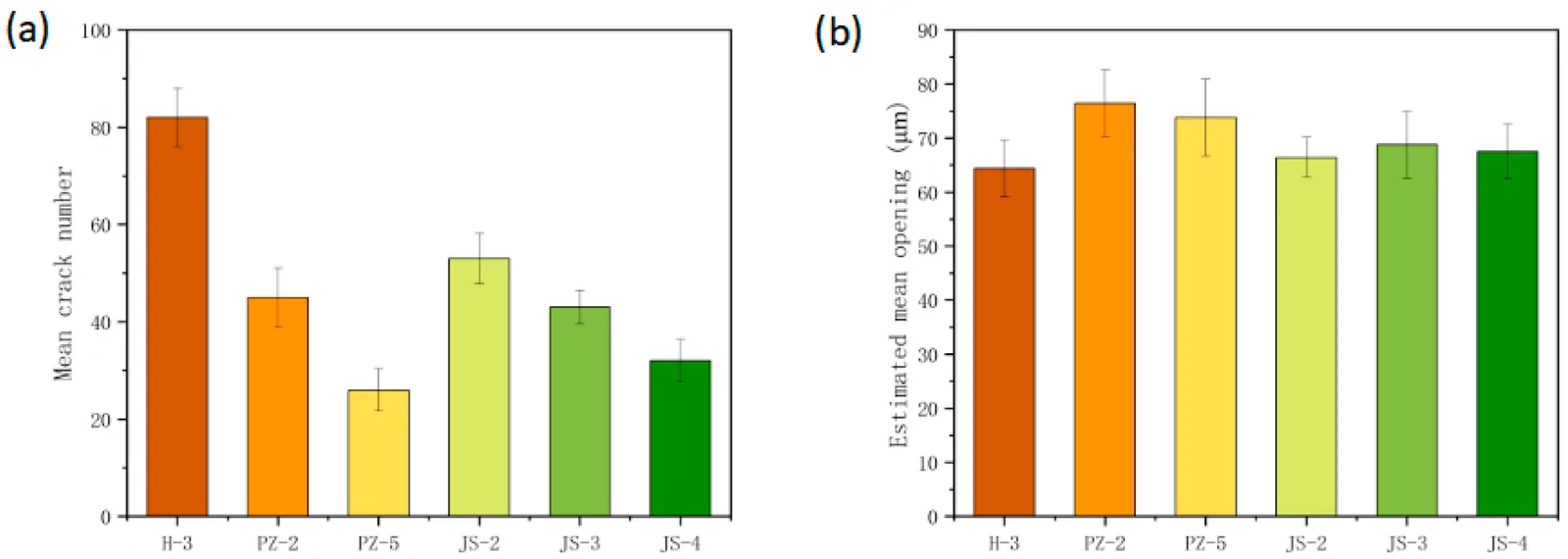
Distributed-cracking indicators at the ultimate tensile state: (**a**) mean crack number and (**b**) strain-based estimated mean crack opening. The dashed line in panel (**b**) indicates the performance criterion.

**Figure 11:**
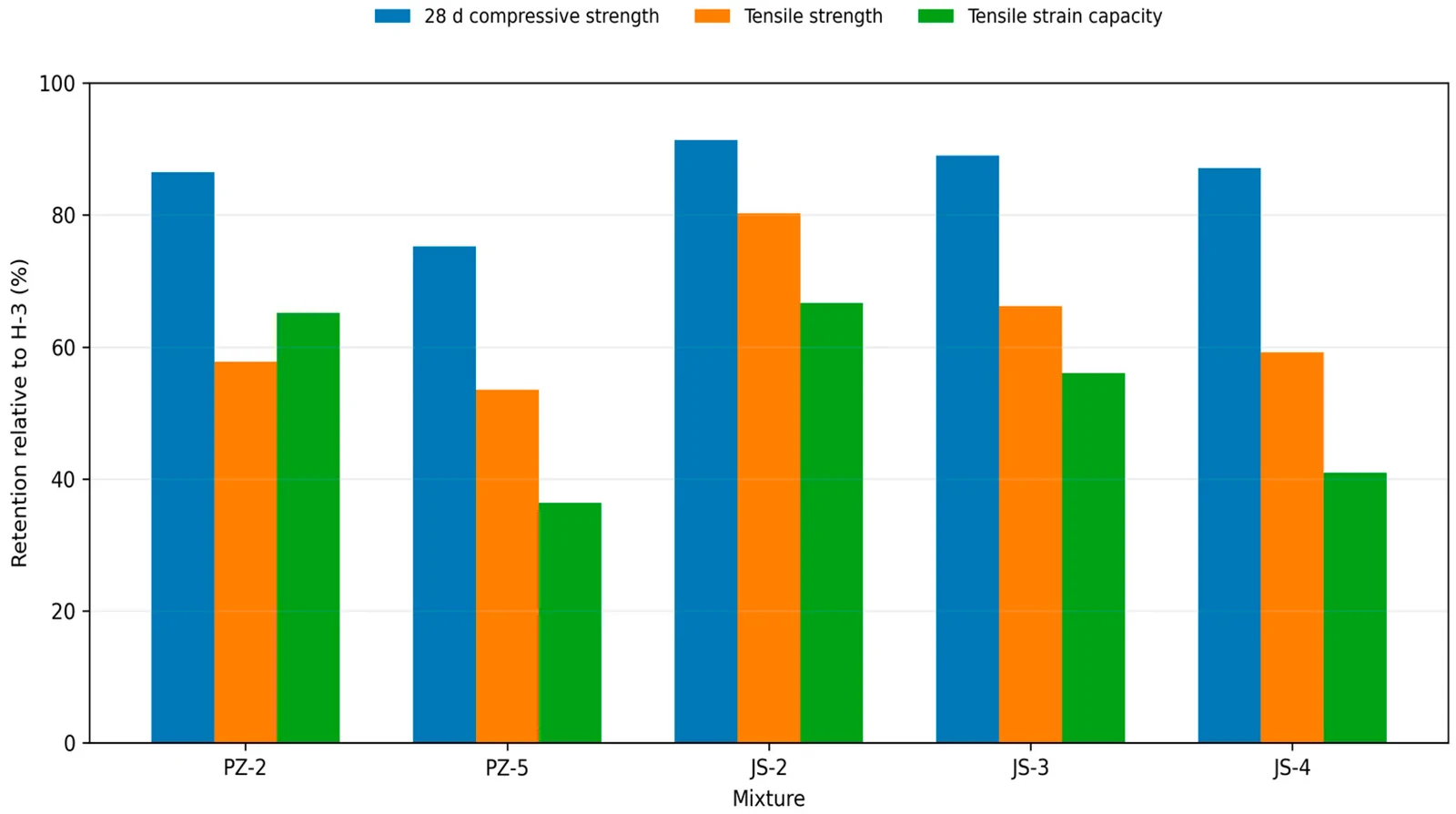
Retention of 28 d compressive strength, ultimate tensile strength, and tensile strain capacity relative to H-3.

H-3 exhibited the highest tensile strength and tensile strain capacity. In Table 10, PZ-2 retained a tensile strain of 4.3%, but its tensile strength decreased to 4.1 MPa; PZ-5 showed reductions to 3.8 MPa in tensile strength and 2.4% in tensile strain. JS-2 retained a tensile strength of 5.7 MPa and a tensile strain of 4.4%, whereas JS-3 and JS-4 exhibited progressively lower tensile properties. All tested mixtures exhibited multiple cracking, with strain-based estimated mean crack openings below 77 μm. The decrease in the number of cracks with increasing modifier dosage indicates a less distributed cracking pattern, whereas the strain-based mean crack opening estimates remained within a narrow range of 64.4–76.4 μm. The representative curves in Figure 8 exhibit repeated load drops associated with sequential matrix cracking and fibre bridging, which supports the qualitative strain-hardening interpretation. Figure 9 and Figure 10 illustrate the loss of tensile capacity together with the accompanying change in crack distribution. The normalized comparison in Figure 11 further demonstrates that considering compressive-strength retention alone would overstate the compatibility of the modifiers. JS-2 retained 91.3% of the reference compressive strength but only 80.3% of the reference tensile strength and 66.7% of the reference tensile strain. PZ-5 retained 75.2%, 53.5%, and 36.4% of the corresponding reference values, respectively, demonstrating the greater sensitivity of tensile deformation capacity in the screened set.

**Table 10:** Ultimate tensile properties and strain-based crack-opening estimates.

Mixture	Tensile Strength (MPa)	Tensile Strain (%)	Mean Crack Number	Estimated Mean Opening (μm)
H-3	7.1	6.6	82	64.4
PZ-1	6.9	5.8	77	60.3
PZ-2	4.1	4.3	45	76.4
PZ-5	3.8	2.4	26	73.8
JS-1	6.2	5.5	69	63.8
JS-2	5.7	4.4	53	66.4
JS-3	4.7	3.7	43	68.8
JS-4	4.2	2.7	32	67.5

### 3.5. Multi-Criteria Selection

**Figure 12:**
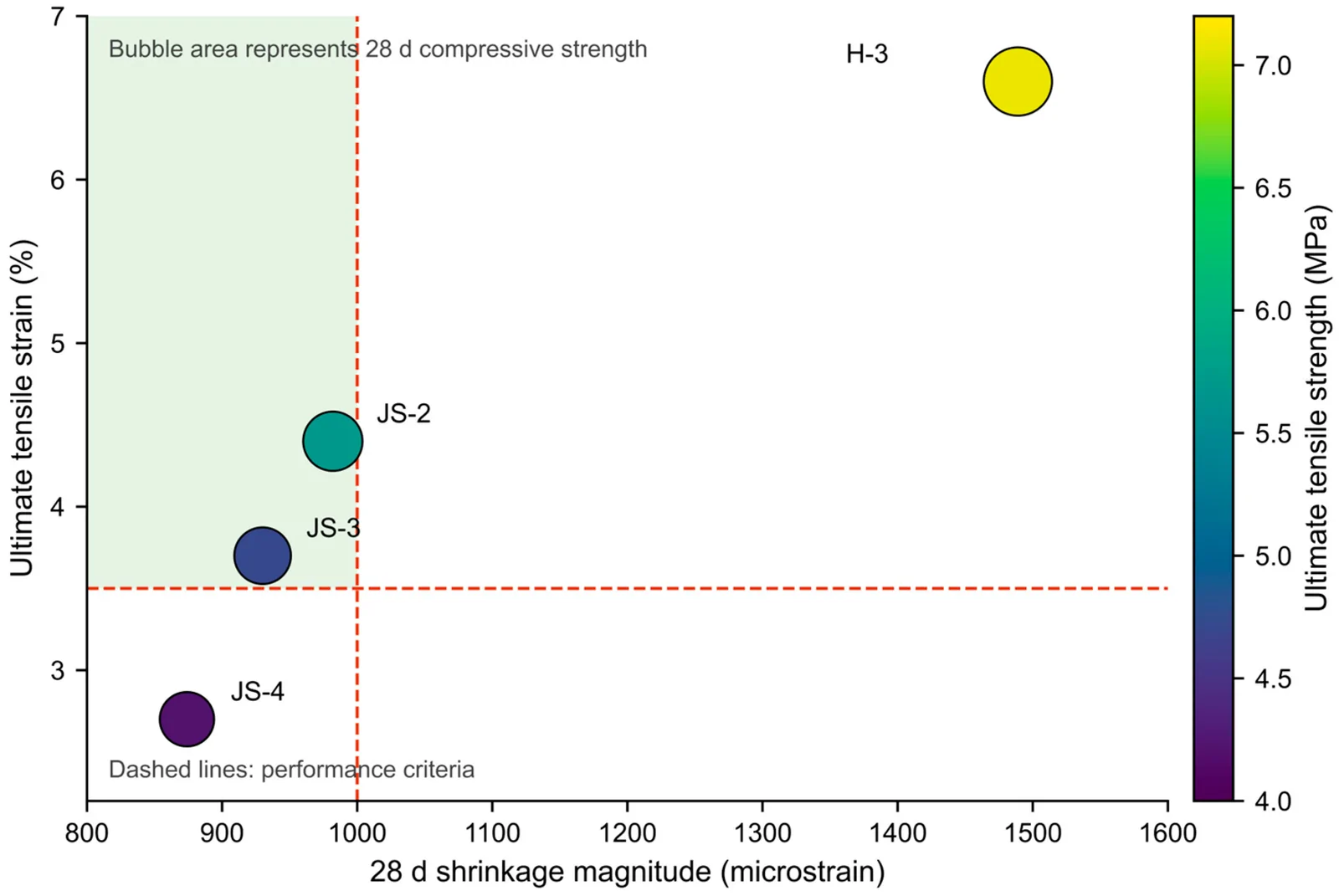
Multi-objective map of shrinkage and tensile performance. Bubble area represents 28 d compressive strength and color represents ultimate tensile strength. Dashed lines show the shrinkage and tensile-strain criteria; the shaded upper-left region satisfies both.

The purpose of the multi-criteria map is to visualize relative margins rather than to provide a rigorous probabilistic acceptance test. Figure 12 visualizes the screened mixtures with tabulated 28 d shrinkage and tensile data. H-3 lies outside the contraction criterion despite its high tensile reserve. JS-4 minimizes contraction but falls below the strain and tensile-strength criteria. JS-3 satisfies the shrinkage and strain criteria but misses the tensile-strength criterion by 0.3 MPa. Based on the mean results, JS-2 lies inside the shrinkage-strain target region and is the only modified mixture that also exceeds 5.0 MPa tensile strength and 70 MPa compressive strength. When the tensile strength requirement is lowered to 3.5 MPa, JS-3 also meets the criteria. The specific data are also shown in Table 11.

**Table 11:** Actual values and margins governing the multi-criteria selection.

Mixture	28 d Length Response (με)	*f_c_*, 28 d (MPa)	*f_tu_* (MPa)	*Ε_tu_* (%)	*w_est_* (μm)
H-3	1489	90.1	7.1	6.6	64.4
JS-2	982	82.3	5.7	4.4	66.4
JS-3	930	80.2	4.7	3.7	68.8
JS-4	874	78.5	4.2	2.7	67.5
PZ-2	+190	77.9	4.1	4.3	76.4
PZ-5	+1880	67.8	3.8	2.4	73.8

## 4. Discussion

### 4.1. Incremental Contribution Relative to Existing ECC Studies

The present results extend existing ECC shrinkage research. Previous studies on PVA-ECC showed that both expansive agents and SRAs affect shrinkage and tensile properties [10]. A subsequent PVA-SHCC comparison found that the balance between MgO and SRA depended on dosage and matrix composition [15]. The current contribution lies in the combination of a 2.5 vol.% PE fibre system, a water-to-binder ratio of 0.27, and a 90.1 MPa reference matrix, together with the explicit tracking of tensile strain, crack number, and strain-based estimates of mean crack opening. In addition, the multi-criteria map makes the selection margin visible, rather than reducing the decision to a binary pass/fail table.

The resulting selection window is specific to the investigated PE-ECC formulation. Gao et al. used a PVA-ECC with w/b between 0.25 and 0.33 and a low-alkali expansive agent [10], while Yuan et al. used a fly-ash-rich PVA-SHCC with coarse silica sand and w/b of 0.30 [15]. Differences in fibre surface chemistry, aggregate skeleton, paste volume, and modifier reactivity can change both shrinkage and tensile response. Accordingly, the selected 1% SRA dosage is interpreted within the present matrix design and adopted performance criteria.

### 4.2. Mechanistic Interpretation of MgO and Liquid SRA Responses

MgO produced a narrow usable range in the free length-change test. At 2%, it reduced the 28 d contraction by only 7.1%, but also caused a reduction in tensile strength of approximately 40% (from 6.9 MPa to 4.1 MPa) and a decrease in ultimate tensile strain of approximately 27% (from 5.8% to 4.3%). At 5% and 8%, the response changed from early contraction to later net expansion. The specimens with 10% and 20% MgO (PZ-3 and PZ-4) exhibited visible bending. The 28 d net expansions were 190 and 1880 με for the 5% and 8% mixtures, respectively. These results reveal a rapid transition from incomplete shrinkage compensation to dimensional instability with increasing MgO dosage.

It has been reported in related studies that MgO expansion originates from hydration to brucite, Mg(OH)_2_. The hydration product occupies a larger solid volume than the original MgO, and its precipitation and crystal growth within capillary space can generate swelling and crystallization pressures. When this deformation develops concurrently with matrix shrinkage, it offsets part of the net contraction [29,30]. The timing is controlled by calcination history, particle microstructure, and reactivity: highly reactive MgO hydrates earlier, whereas less reactive MgO can continue hydrating after the cementitious matrix has stiffened and shift expansion to later ages [29,30]. Moisture availability further regulates the hydration degree [15,16], while curing temperature and matrix restraint affect the magnitude and timing of expansion [17,18].

According to Liu et al. and Zhao et al. [16,17,18], at a suitable dosage, brucite formation can occupy available pore space and compensate for contraction. At an excessive dosage, or when hydration is delayed or spatially nonuniform, the resulting expansive eigenstrain may exceed the accommodation and restraint capacity of the hardened matrix, promoting internal tensile stress, microcracking, curvature, and loss of dimensional soundness. Expansion-model studies also show that the balance between pore-space accommodation and crystallization pressure changes with MgO reactivity [29,30]. The transition to net expansion and the bending observed here are consistent with this mechanism. In ECC, matrix fracture resistance and fibre-bridging capacity must also remain compatible for strain hardening [7,10]. The lower tensile strength and strain of PZ-2 and PZ-5 suggest a disturbance in this matrix–fibre balance. MgO-ECC studies likewise show that dosage, MgO reactivity, and supplementary cementitious materials jointly control expansion and composite performance [31]. These mechanisms are literature-based hypotheses; no XRD, thermogravimetric, calorimetric, microscopic, or interfacial measurements were performed in the present study to verify hydration products or fibre–matrix changes. Free drying length change combines processes that cannot be individually separated by this test. Autogenous shrinkage and restraint-induced stress were not measured, and a free-expansion value must not be equated with crack-free behavior in a bonded repair layer.

The liquid SRA produced a smoother dosage response. At an SRA dosage of 0.5%, the shrinkage of the specimens was improved to some extent, decreasing from 1489 με to 1265 με; however, it still exceeded the screening limit of 1000 με, and this reduction was accompanied by a sacrifice in tensile performance. Increasing dosage from 1% to 3% lowered the 28 d shrinkage magnitude from 982 to 874 με, while flow diameter changed by only 3 mm. However, tensile strength declined from 5.7 to 4.2 MPa and tensile strain from 4.4% to 2.7%. In an ECC, a weaker matrix or a change in fibre–matrix interfacial stress can reduce the bridging reserve available for stable multiple cracking [7,10]. The concurrent decline in tensile capacity and crack number with increasing SRA dosage is consistent with this coupled mechanism. In the present formulation, the additional 108-μm reduction obtained by increasing SRA from 1% to 3% did not compensate for the loss of tensile reserve under the adopted performance criteria. The literature describes many SRAs as polyol- or polyether-based formulations that can lower pore-solution surface tension and thereby lower capillary pressure during drying and self-desiccation; examples include glycol and glycol-ether chemistries [14]. They can also modify pore-solution chemistry and retard cement hydration, which may slow matrix strength development [32]. The effects depend on chemical identity, molecular weight, concentration, moisture conditions, and interactions with other admixtures. The exact formulation and active content of the SRA used here were not available in the submitted material, so this explanation remains generic and cannot establish the mechanism of the particular commercial product.

### 4.3. Why Shrinkage Minimization Alone Is Insufficient

A repair mortar is expected to accommodate substrate deformation while maintaining stress transfer across distributed cracks. JS-4 produced the smallest contraction, but its tensile strain was 38.6% lower than JS-2 and below the tensile-strain criterion. Flexural strength alone would not have revealed this loss, because all SRA mixtures retained more than 15.7 MPa at 28 d. Direct tension was therefore the discriminating test. JS-2 retained 80.3% of the reference tensile strength and 66.7% of its tensile strain while reducing the shrinkage magnitude by 34.0%.

The crack-opening calculation provides a comparative index of how gauge deformation was partitioned among the counted cracks. The 64.4–76.4 μm, together with the declining crack number, indicate that the loss of tensile strain was expressed mainly through fewer cracks rather than a proportional increase in estimated mean opening. The crack-distribution results therefore reinforce the need to evaluate tensile deformation capacity together with shrinkage and strength.

### 4.4. Implications for Hydraulic-Concrete Repair

JS-2 is expected to exhibit a well-balanced performance in hydraulic repair applications, which indicates that this material represents a promising candidate matrix combining high strength with favorable deformability. However, its actual repair performance depends on substrate stiffness, surface conditions, overlay thickness, curing conditions, restraint state, and hydraulic exposure conditions. These factors govern the stress distribution, interfacial integrity, and mass transport properties of the repair system. Since bond performance on wet substrates, impermeability pressure resistance, and wet-dry cycling tests were not addressed in this study, further investigations incorporating these tests and analyses are planned to provide a basis for validating the practical application of this material.

## 5. Conclusions and Summary

1. The unmodified high-strength PE-ECC combined 90.1 MPa 28 d compressive strength, 7.1 MPa tensile strength, and 6.6% tensile strain, but its 28 d drying shrinkage reached 1489 με.
2. MgO reduced flow diameter and produced a narrow dimensional-control window. A 2% dosage reduced the 28 d contraction to 1384 μm, whereas 5% and 8% changed from early contraction to 28 d net expansions of 190 and 1880 μm, respectively. Specimens with 10% and 20% MgO showed visible bending.
3. Liquid SRA retained flow diameters of 182–185 mm and reduced 28 d shrinkage to 982, 930, and 874 με at 1%, 2%, and 3%, respectively. The shrinkage benefit increased with dosage, but tensile strength, tensile strain capacity, and crack number decreased.
4. Within the investigated threshold range, JS-2 is the most robust candidate, which retained 82.3 MPa compressive strength, 5.7 MPa tensile strength, 4.4% tensile strain, and an estimated mean crack opening of 66.4 μm. But its choice depends on the adopted performance limits. This is a conditional screening result, not evidence of statistical superiority or a universally optimal dosage. The selection of JS-2 (1% SRA dosage) is based on the specific polyether-type SRA investigated, and other SRA chemistries may require re-evaluation.
5. Based on the experimental results of this study, the selected mixture can be considered as a candidate material for future application in hydraulic concrete repair, particularly for thin repair layers and localized surface restoration where dimensional compatibility and tensile deformability are of critical importance.

## Figures and Tables

**Table 5:** Stage I mixture matrix, measurements, and screening outcomes.

Mixture	Modifier	Flow (mm)	Length Change	Strength	Stage I Outcome
H-3	Reference	185	3, 7, 14, 28 d	1, 7, 28 d	Benchmark
PZ-1	2% MgO	182	3, 7, 14, 28 d	1, 7, 28 d	Stable; 1384 με contraction
PZ-2	5% MgO	175	3, 7, 14, 28 d	1, 7, 28 d	Net-expansion transition
PZ-5	8% MgO	172	3, 7, 14, 28 d	1, 7, 28 d	Pronounced net expansion
PZ-3	10% MgO	169	Screened to 28 d	Not continued	Visible bending
PZ-4	20% MgO	160	Screened to 28 d	Not continued	Visible bending
JS-1	0.5% SRA	185	3, 7, 14, 28 d	1, 7, 28 d	1265 με contraction
JS-2	1% SRA	185	3, 7, 14, 28 d	1, 7, 28 d	982 με contraction
JS-3	2% SRA	183	3, 7, 14, 28 d	1, 7, 28 d	930 με contraction
JS-4	3% SRA	182	3, 7, 14, 28 d	1, 7, 28 d	874 με contraction

**Table 7:** Performance criteria used for multi-criteria mixture selection.

Indicator	Criterion	Engineering Purpose
28 d shrinkage magnitude	≤1000 με	Limit free contraction in thin repair layers
28 d compressive strength	≥70 MPa	Maintain a high-strength repair matrix
28 d flexural strength	≥10 MPa	Maintain bending resistance
Ultimate tensile strength	≥5.0 MPa	Maintain fibre-bridging stress capacity
Ultimate tensile strain	≥3.5%	Maintain deformation compatibility
Estimated mean crack opening	≤100 μm	Promote distributed cracking

## Data Availability

The original contributions presented in this study are included in the article. Further inquiries can be directed to the corresponding author.

## Abbreviations

ECC, engineered cementitious composite; PE, polyethylene; PE-ECC, polyethylene-fibre engineered cementitious composite; SRA, shrinkage-reducing admixture; MgO, magnesium oxide; w/b, water-to-binder ratio; PCA, polycarboxylate admixture; RH, relative humidity; XRD, X-ray diffraction; SEM, scanning electron microscopy.
